# Perspectives of pediatric oncologists on referral for CAR-T therapy: a mixed methods pilot study

**DOI:** 10.1093/jncics/pkae063

**Published:** 2024-07-30

**Authors:** Anurekha G Hall, Devan M Duenas, Jenna Voutsinas, Qian Wu, Adam J Lamble, Elizabeth Gruber, Benjamin Wilfond, Julie R Park, Anurag K Agrawal, Jonathan M Marron

**Affiliations:** Division of Hematology and Oncology, University of Washington, Seattle Children’s Hospital, Seattle, WA, USA; Clinical Research Division, Fred Hutchinson Cancer Center, Seattle, WA, USA; Treuman Katz Center for Pediatric Bioethics and Palliative Care, Seattle Children’s Research Institute, Seattle, WA, USA; Clinical Research Division, Fred Hutchinson Cancer Center, Seattle, WA, USA; Clinical Research Division, Fred Hutchinson Cancer Center, Seattle, WA, USA; Division of Hematology and Oncology, University of Washington, Seattle Children’s Hospital, Seattle, WA, USA; Seattle Children’s Therapeutics, Seattle, WA, USA; Treuman Katz Center for Pediatric Bioethics and Palliative Care, Seattle Children’s Research Institute, Seattle, WA, USA; Department of Oncology, St Jude Children’s Research Hospital, Memphis, TN, USA; Division of Hematology and Oncology, UCSF Benioff Children’s Hospital Oakland, Oakland, CA, USA; Department of Pediatric Oncology, Dana-Farber Cancer Institute, Boston, MA, USA; Center for Bioethics, Harvard Medical School, Boston, MA, USA

## Abstract

**Background:**

Receipt of chimeric antigen receptor T-cell (CAR-T) therapy at an institution different from the primary oncologist’s institution is a complex, multistep process. Referral by oncologists plays an important role in the process but may be susceptible to bias.

**Methods:**

Oncologists who previously referred patients for CAR-T therapy at 5 pediatric hospitals were sent surveys by email exploring their CAR-T referral practices. Descriptive statistics were generated, and multivariate analyses examined associations among oncologist characteristics, familiarity with CAR-T therapy, and referral practices. We conducted semistructured interviews with a subset of participants and used thematic analysis to code transcripts.

**Results:**

Sixty-eight oncologists completed the survey; 77% expressed being “very familiar” with CAR-T therapy. Hispanic oncologists and oncologists at institutions with 50 or fewer new diagnoses per year were more likely to identify as less familiar with CAR-T therapy (odds ratio [OR] = 64.3, 95% confidence interval [CI] = 2.45 to 10 452.50, *P* = .04 and OR = 24.5, 95% CI = 3.3 to 317.3, *P* = .005, respectively). In total, 38% of respondents considered nonclinical features (compliance, social support, resources, insurance, language, education, and race or ethnicity) influential in referral decisions. Oncologists who were Hispanic and oncologists who had been practicing for 20 or more years were more likely to consider these features significantly influential (OR = 14.52, 95% CI = 1.49 to 358.66, *P* = .04 and OR = 6.76, 95% CI = 1.18 to 50.5, *P* = .04). Nine oncologists completed in-depth interviews; common themes included barriers and concerns regarding CAR-T therapy referral, the value of an established relationship with a CAR-T therapy center, and poor communication after CAR-T therapy.

**Conclusions:**

Nearly 40% of oncologists consider nonclinical features significantly influential when deciding to refer patients for CAR-T therapy, raising concern for bias in the referral process. Establishing formal partnerships with CAR-T therapy centers may help address physician barriers in referral.

Chimeric antigen receptor T-cell (CAR-T) therapy has revolutionized the treatment of relapsed-recurrent pediatric B-cell acute lymphoblastic leukemia (B-ALL), with more than 80% of patients achieving complete remission and approximately 50% maintaining that remission 12 months after infusion (1,2). Although tisagenlecleucel remains the only US Food and Drug Administration–approved CAR-T product for patients younger than 18 years of age, more than 100 pediatric CAR-T clinical trials are currently in progress (3,4). Due to high manufacturing cost, the need for certified manufacturing facilities, and the need for advanced clinical expertise, CAR-T products are available at a limited number of institutions and therefore are not equitably accessible to all patients. Transportation and distance are well-established barriers to health-care access for patients of low socioeconomic status (SES) (5), and patients with low SES, patients who have public insurance, and patients from historically marginalized racial and ethnic groups are all less likely to receive CAR-T therapy (6,7). Recent evidence demonstrates similar outcomes in patients able to receive CAR-T therapy, regardless of race, ethnicity, or SES, further highlighting the importance of access (8,9).

Receipt of CAR-T therapy is a complex, multistep process for patients (Figure 1), with additional obstacles for patients who must travel to an institution different from that of their primary oncologist to access the therapy. Referral by oncologists plays a critical role in this process but may be susceptible to bias. Moreover, numerous therapeutic options are now available for relapsed-recurrent pediatric B-ALL, making analysis of “optimal” referral practices quite challenging (10). Oncologists have previously identified lack of social support and insurance coverage as determinants of their referral practices for hematopoietic stem cell transplant (11,12). Protocol complexity, limited time and resources, and lack of educational resources have been described as additional barriers that may discourage clinicians from referring patients to clinical trials (13-15), and although clinical trials are central to the practice of pediatric oncology, pediatric oncologists note similar barriers (16,17). The importance of these nonclinical factors and the possibility of unique barriers have not been studied in the context of CAR-T referral practices. Through the National Institutes of Health–funded Consortium for Pediatric Cellular Immunotherapy (CPCI), we conducted a mixed-methods study to explore variations in pediatric oncologists’ self-reported referral practices and, secondarily, to assess whether evidence of bias exists in referral practices. We hypothesized that oncologists consider factors aside from clinical status and medical eligibility when deciding whether to refer patients for CAR-T therapy.

**Figure 1.**
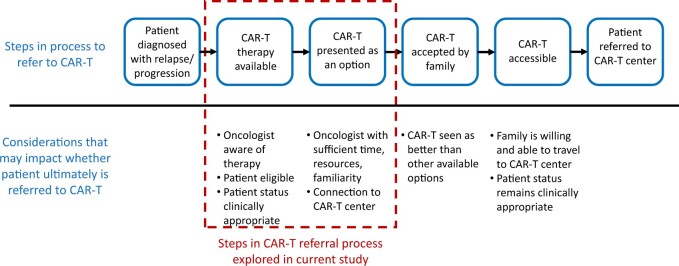
Process to refer patients to CAR-T. CAR-T = chimeric antigen receptor T cell.

## Methods

As previously described (6), the CPCI includes 5 sites, all of which are leading referral centers for pediatric CAR-T therapy: Seattle Children’s Hospital, University of California San Francisco Benioff Children’s Hospitals, Children’s Hospital of Los Angeles, Children’s Hospital of Colorado, and Children’s National Hospital. This study, a collaboration among these sites, was approved by institutional review boards at each institution. We used a convenient sample of clinicians who had previously referred a patient to a CPCI site for a CAR-T clinical trial and queried their CAR-T referral practices, both for clinical trial and commercial products, with the expectation that referral practices are largely similar when a physician must refer to an outside institution.

### Survey development

Survey items were developed by study team members with expertise in pediatric oncology, immunotherapy, survey methodology, and bioethics. The survey (Supplementary Material, available online) included novel items assessing oncologist demographics, institution characteristics, familiarity with CAR-T therapy, and factors perceived to be influential in deciding to refer patients for CAR-T therapy, informed by relevant literature in this field (11,12,15,16,18). The CPCI Patient Advocacy Committee reviewed and approved all survey items, which were then pilot-tested with oncologists outside the study team.

Respondents were queried regarding their familiarity with CAR-T therapy and their comfort providing information about CAR-T therapy to families. To examine referral decisions and assess explicit bias, respondents were presented with 13 patient-level, family-level, or institution-level factors and asked to rate how influential each factor was in their decision to refer. Nonclinical patient-level and family-level factors (family resources, social support, insurance, compliance, language, education, and race or ethnicity) were categorized as “nonclinical features” to evaluate the role these factors were perceived to have in physician referral. These specific factors were adapted from prior literature (19) and identified ex ante because of a lack of clinical or scientific rationale for their inclusion in referral decisions, thus raising the possibility of bias.

To further query biases, the survey included 4 iterations of a hypothetical scenario involving a pediatric patient with relapsed B-ALL. Respondents were asked to rate the favorability of each hypothetical patient for CAR-T therapy, how likely they were to refer each patient for CAR-T therapy, and how likely the patient and family were to choose to proceed with CAR-T therapy. Scenarios described patients with similar clinical situations but with different race (Black vs White) or parent education level (college degree vs incomplete high school).

### Survey administration

Each site sent emails to oncologists who had previously referred patients with B-ALL to 1 of the CPCI sites for CAR-T clinical trials. The email included an electronic Research Electronic Data Capture survey link. Respondents received a $20 gift card.

### Survey analysis

Descriptive statistics were performed to depict features of the cohort and participant perspectives on and experiences with CAR-T referral. Missing values were excluded from percentage calculations and relevant analyses. Binary outcomes included 1) familiarity with CAR-T therapy (“very familiar” vs “somewhat familiar”) and 2) a composite outcome of those influenced vs not influenced by nonclinical features. For this composite outcome, participants who rated 1 or more of the 7 selected nonclinical features as “significantly influential” were coded as “influenced” (with all others coded as “not influenced”). Univariate and multivariable logistic regression was conducted to assess associations between these outcomes and covariates of interest. Variables selected in the multivariable analyses were based on scientific interest (not statistical testing from univariate analysis). A linear mixed-effects model was used to compare responses across the 4 patient scenarios for each scenario question. Odds ratios (ORs), 95% confidence intervals (CIs), and *P* values were calculated. All analyses were performed using R, version 4.1.3, statistical software (R Foundation for Statistical Computing, Vienna, Austria).

### Interviews

All survey respondents were invited to participate in a follow-up semistructured interview to provide a more in-depth understanding of a subset of respondents’ perspectives and experiences. The interview guide (Supplementary Material, available online) was collaboratively developed by the research team based on existing literature along with early aggregated survey responses (11-13,15,16). Interview questions queried participants’ experiences referring patients for CAR-T therapy through a clinical trial, obstacles and barriers experienced throughout the process, and facilitators of the referral process. The CPCI Patient Advisory Committee reviewed and approved the interview guide. Interviews were conducted in English by 1 study team member (A.G.H.), recorded using a secure Zoom session (Zoom Video Communications, San Jose, CA), professionally transcribed, and deidentified. Verbal consent was obtained at the time of the interview. Interview participants received $50 gift cards.

Interview transcripts were uploaded to Dedoose (dedoose.com) for data management and thematic analysis (20). The study team took a hybrid approach to analysis, using a deductive approach to first develop a qualitative codebook based on domains of the interview guide and informed by relevant literature (13,15,18). This initial codebook was tested to evaluate reliability and identify additional domains, with additional inductive codes added following review by the study team (21-23). When the codebook was finalized and approved, all transcripts were systematically coded by 2 coders (A.G.H. and D.M.D.), with discrepancies reviewed and resolved with the support of the entire study team. After all data were coded, 1 coder (A.G.H.) created code summaries, and results were reviewed by the study team to identify key themes and other notable findings. Survey and interview data were not linked, given that responses to both were anonymous.

## Results

### Survey

The survey was sent by email to 155 referring oncologists; 68 (44%) responded. Some survey items were not completed by all respondents. Twenty-six respondents (41%) practiced at centers with more than 100 new pediatric oncology diagnoses per year (Table 1). Forty-four (90%) respondents identified as non-Hispanic; 34 (69%) identified as White, 9 (18%) as Asian, 1 (2%) as Black, and 5 (10%) as other. Thirteen (26%) had been in practice for more than 20 years. Oncologists felt that the most common barriers patients faced in accessing CAR-T therapies were clinical factors, family resources, insurance status, and family social support.

**Table 1.** Characteristics of referring physicians and institutions

Characteristic	**No. (% of responses)**
**Physician characteristics (N = 68)^a^**	
Age	
<40 y	5 (10.0)
40-60 y	36 (72.0)
>60 y	9 (18.0)
Sex	
Female	32 (65.3)
Male	17 (34.7)
Race	
Asian	9 (18.4)
Black	1 (2.0)
White	34 (69.4)
Other	5 (10.2)
Ethnicity	
Hispanic	5 (10.2)
Non-Hispanic	44 (89.8)
Years in practice	
<5 y	3 (6.0)
5-10 y	12 (24.0)
11-20 y	22 (44.0)
>20 y	13 (26.0)
**Institution characteristics**	
No. of pediatric oncologists at center	
<5	13 (20.3)
5-10	30 (46.9)
>10	21 (32.8)
No. of new pediatric oncology diagnoses/y at center	
<25	5 (7.8)
25-50	9 (14.1)
51-100	24 (37.5)
>100	26 (40.6)
**CAR-T experience in the prior 12 mo**	
How many patients were potentially eligible for CAR-T therapy?	
0-1	7 (10.9)
2-3	23 (35.9)
>3	34 (53.1)
What percentage of eligible patients were referred for CAR-T therapy?	
0%	6 (9.4)
1-50%	8 (12.5)
51-99%	35 (54.7)
100%	15 (23.4)
How many patients declined referral?	
0-1	55 (85.9)
2-3	8 (12.5)
>3	1 (1.6)
How familiar are you with CAR-T therapies?	
Not familiar	0 (0.0)
Somewhat familiar	15 (23.4)
Very familiar	49 (76.6)
How comfortable are you providing information about CAR-T therapy to your patients and their families?	
Not very comfortable	1 (1.6)
Somewhat comfortable	14 (21.9)
Very comfortable	49 (76.6)

a Total numbers may not sum to 68 due to item nonresponse. CAR-T = chimeric antigen receptor T cell.

Forty-nine (77%) respondents were “very familiar” with CAR-T therapy. Similarly, 77% were “very comfortable” providing information about CAR-T therapy to patients. In multivariate analysis, Hispanic oncologists (OR = 64.3, 95% CI = 2.45 to 10 452.47, *P* = .04) and oncologists at institutions with fewer than 50 new diagnoses per year (OR = 24.5, 95% CI = 3.29 to 317.3, *P* = .005) were more likely to identify as being less familiar with CAR-T therapy (Table 2).

**Table 2.** Oncologist characteristics associated with self-identifying as less familiar with chimeric antigen receptor T-cell therapy

Characteristic	**Somewhat familiar, No. (n = 15)** ^a^	**Very familiar, No. (n = 49)** ^a^	Odds ratio (95% confidence interval)	*P*
Sex				
Female	11	21	—	—
Male	1	16	0.13 (0.01 to 1.13)	.11
Ethnicity				
Non-Hispanic	9	35	—	—
Hispanic	3	2	64.3 (2.45 to 10 452.5)	.04
Race				
White	9	25	—	—
Non-White	3	12	0.12 (0.00 to 1.48)	.17
Years in practice				
≤10	3	12	—	—
11-20	6	16	5.97 (0.49 to 138.1)	.20
>20	3	10	18.7 (1.10 to 729.04)	.07
No. of new diagnoses/y				
>50	6	44	—	—
≤50	9	5	24.5 (3.3 to 317.3)	.005

a Total numbers vary due to item nonresponse.

Fifty-one participants completed all survey items regarding the 4 patient scenarios (data not shown). Of these 51 participants, 40 (78%) rated the White patient with college-educated parents as a “very favorable” CAR-T therapy candidate, and 36 (71%) reported the same for the White patient with parents who had not received a high school education (*P* = .02). No other statistically significant differences were observed across scenarios for pairwise comparisons.

**Figure 2.**
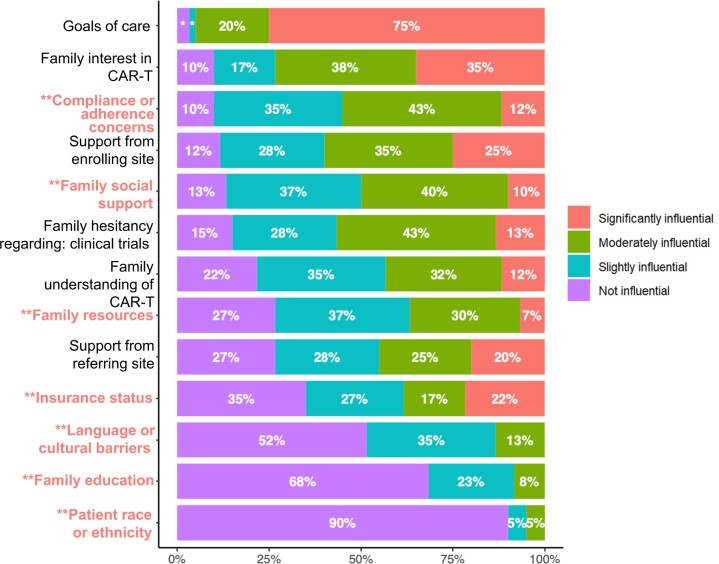
Factors considered influential when deciding to refer a patient for CAR-T therapy. CAR-T = chimeric antigen receptor T cell. ^*^ Goals of care: 3% not influential and 2% slightly influential. ^**^ Factors included in the composite outcome as nonclinical features.

Fourteen respondents (22%) estimated that fewer than half of eligible patients at their institution were referred for CAR-T therapy. Factors respondents self-identified as most influential in their decision to refer included family goals of care (75%), family interest in CAR-T therapy (35%), support from enrolling site (25%), insurance (22%), and family resources (20%) (Figure 2). Twenty-three (38%) respondents considered nonclinical features to be significantly influential in referral decisions. In multivariate analysis, Hispanic oncologists (OR = 14.52, 95% CI = 1.49 to 358.66, *P* = .04) and oncologists who had been practicing for at least 20 years (OR = 6.76, 95% CI = 1.18 to 50.5, *P* = .04) were more likely to consider nonclinical features significantly influential (Table 3).

**Table 3.** Oncologist characteristics associated with finding nonclinical features influential

Characteristic	**Not significantly influential, No. (n = 37)** ^a^	**Significantly influential, No. (n = 23)** ^a^	Odds ratio (95% confidence interval)	*P*
Sex				
Female	20	12	—	—
Male	9	8	1.98 (0.51 to 8.18)	.33
Ethnicity				
Non-Hispanic	28	16	—	—
Hispanic	1	4	14.5 (1.49 to 358.7)	.04
Race				
White	21	13	—	—
Non-White	8	7	0.96 (0.20 to 4.21)	.95
Years in practice				
≤10	10	5	—	—
11-20	15	7	1.51 (0.30 to 8.96)	.63
>20	5	8	6.76 (1.18 to 50.5)	.04
No. of new diagnoses/y				
>50	30	16	—	—
≤50	7	7	1.65 (0.32 to 8.23)	.54

a Total numbers vary due to item nonresponse.

### Interviews

Fifteen respondents expressed interest in participating in interviews, with 9 (60%) ultimately completing interviews. Findings centered on 3 major themes: barriers and concerns, the value of an established relationship with a CAR-T therapy center, and poor communication after CAR-T therapy (Table 4).

**Table 4.** Themes from interviews with referring physicians^a^

**Barriers and concerns**	
Financial hardships	“There’s the financial part of it sort of directly involved with covering the procedure, but then there’s all those out-of-pocket costs that they have to deal with living out of state and being away from your support, sometimes needing to take time off from work so your income is disrupted.” “What is a challenge is also the amount of poverty in our state. Sometimes, we have families that don't have cars, so it's a challenge to then get them to appointments here much less to [CAR-T therapy site city].”
Challenges of leaving home site	“A family’s ability to be away, not only just having a place to stay, but also, what other children do they have? How old are they? Who's going to be at home? What kind of family support do they have? Who can help keep all that going while they’re away? At the end of the day, the family has to weigh all of that.” “Whether a family is going to tolerate being away from home, especially if it involves being far away in a different city … their support systems are usually at home. And for a child, it’s all about being able to be in their own room and sleep in their own bed, those kinds of things; all of that goes away when they get referred somewhere else.” “One of the other interesting things that comes up is the transfer of institutions. They feel very comfortable, very at home here with their nurses and their doctors, and they’re going to a larger institution. And the doctors and nurses are great there, but sometimes but there isn’t as individual of a touch there.”
No insurmountable barriers	“We have not had [finances] be an absolute barrier. Families make it happen. Despite what happens after that, after the downfall, families make it happen, and if we’re in a situation where we need CAR-T therapy, then they get out there to do it.” “If there is anything that offers hope, then most [families] are willing to travel and work things out [to get] other family members to help with other kids.” “Families will go to a lot of effort to make something happen if they think their child’s going to benefit from it.”
Concern for long-term impacts	“I worry more about a year or two years down the road. What happens to these families that don’t have as many resources? We can pay an electric bill or we can pay a mortgage payment, but we can only do that so many times. And for somebody especially that has relapsed disease, then you’re chewing up a lot of resources, and I'm sure it’s broken up a lot of marriages and I worry about the other siblings.” “Most families for a child that they think is going to die, they rally. They just do incredible things to make it happen, but there’s probably payback later.” “Parents are stuck in jobs they didn’t want to stay in because of health-care insurance or they haven’t been promoted like they probably would have because they were gone so much. So I think there's economic toll, but just that toll on personal growth and development too.”
**Established relationship with a CAR-T therapy center**	
Source of reliable information	“Honestly we’re very dependent on you to sort of say, ‘We should go to transplant straight away,’ or ‘We should do CAR-T cells and then transplant.’ So I don’t feel like we’re making the final decision for CAR-T cells quite honestly.” “That’s why I feel pretty educated on CAR-Ts even though I don’t do them myself, because I’ve heard him give the CAR-T talk quite a few times”
Easier logistics and care coordination	“We do have existing relationships within these centers, right, that take care of [our institution’s patients] all the time, so it makes the care coordination a little bit easier.” “It is the hard work of every institution, but the fact that the [CAR-T therapy center] coordinators are given the time and money and ability to coordinate everything, that it is so friction-free for me.”
Ability to share care between institutions	“I [say], ‘This is a partnership. I will be taking care of you through the bridging period. This is a thing where we're going to do it together’ … and it helps that I know the doctors there real well, and so I tell them, ‘I've worked with them for over a decade, and I know them and I trust them.’” “I knew I was headed to a small county program … and I really was wondering, ‘Could I even offer these things, or do I just have to accept that I can’t?’ But we’ve been able to, thanks to everybody’s flexibility and desire to get these patients on study and treated. I would have been happy just sending them and never seeing them again, but the fact that [the CAR-T therapy center has] been able to offer me studies where I can have my patients back and still make sure they get study labs and good follow-up and the care that they need has been amazing.” “I will admit I’m not excited to put patients on [other] studies … when I’ve referred patients for a [different] study, I know I’m never going to see them again. And that’s a hard sell, both for me and for the patient. The patient is not thrilled to know that after this conversation they’ll never see me again. And it feels like a dump. It feels like I'm getting rid of them. And they don’t feel ready.”
**Poor communication during and after CAR-T therapy**	
	“Once they go inpatient, then it’s like a black hole.” “It would be not too much to ask that the physician pick up the phone and communicate back to us… . We get hundred pages of faxes. We don’t want a single page. We really don’t want a single page. Don’t kill trees, but just pick up the phone one time and say, ‘I'll give you full sign-out,’ and we’ll be thrilled… . . And it leaves a good taste in your mouth to send another kid back to you, instead of thinking like, ‘Oh, I’ll send it and then nobody will call me back.’” “It’s very challenging when a patient comes back to us. We have to do a lot to get our patients out there and sent a lot of summaries and information and etc. They want all the details, right, as they should. But to come back to us, we get, a lot of times, we just don’t get any information, and we’re trying to piece things together as to why were you in the [intensive care unit] as much?”

a CAR-T = chimeric antigen receptor T cell.

### Barriers and concerns

Common barriers described by interviewees fell into 2 categories: 1) financial barriers and 2) challenges of families leaving their home institution. Interviewees noted that these barriers could be mitigated by social work support, local housing at the CAR-T therapy center, and charitable foundation funding. Though all participants acknowledged the presence of barriers, several explicitly mentioned that none were insurmountable, specifically noting that oncology care teams and families would do whatever it takes to provide patients access to CAR-T therapy. One oncologist noted, “If there is anything that offers hope, then most families are willing to travel and work things out [to get] other family members to help with other kids.” Finally, some oncologists expressed concern for long-term impacts on families, noting that although social work support may be able to provide short-term assistance (eg, mortgage payment, housing, electric bills), they remained concerned about the long-term financial consequences and the psychosocial toll on families.

### Established relationship with a CAR-T therapy center

Several oncologists discussed the value of an established relationship with the CAR-T therapy center in the referral process and identified several mechanisms by which this relationship facilitated referral. Referring oncologists found the CAR-T therapy center to be a source of reliable information, with one noting, “We’re very dependent on [the CAR-T therapy center] to say, ‘We should go to transplant straight away’ or ‘We should do CAR-T, and then transplant.’” In addition, oncologists endorsed easier care coordination within these established relationships that made the process “friction free” for them. Finally, oncologists identified the ability to share care between institutions as important to them and their patients. One interviewee commented that “the fact that [CAR-T therapy centers] have been able to offer me studies where I can have my patients back and still make sure they get study labs and the care they need has been amazing” and specifically called out the comparison to other referrals, where “I know I’m never going to see them again, and that’s a hard sell for both me and the patient.”

### Poor communication after CAR-T therapy

A final theme that emerged in interviews was poor communication after CAR-T. Several participants noted that despite the amount of coordination that occurs during referral and prior to CAR-T, there was inadequate communication from the CAR-T center after patients received CAR-T. One oncologist noted that an experience with poor communication led to a reluctance to refer additional patients and felt that good communication “leaves a good taste in your mouth to send another kid back to [CAR-T center].”

## Discussion

Despite the important role that physicians play in the referral process, data about CAR-T therapy referral practices within pediatric oncology are limited. This study is among the first to describe clinician perspectives and experiences on referral. We found that nearly 40% of oncologists consider patient and family nonclinical features (compliance, social support, resources, insurance, language, education, race and ethnicity) significantly influential when deciding whether to refer patients for CAR-T therapy. Often referred to in the literature as *socioeconomic characteristics* (19), these factors were frequently considered barriers to referral in this cohort. This finding may exacerbate existing disparities within pediatric oncology (6,24-27) and raises concern that otherwise-eligible patients may not be referred because of such factors. Oncologists who have been practicing for more than 20 years were more likely to find these factors influential, possibly in part due to less familiarity with current advanced therapies and less awareness of resources available for patients, though this study could not pinpoint the drivers behind this finding. Hispanic oncologists were also more likely to find these factors influential, and although this finding was statistically significant, the small number of Hispanic participants precludes any meaningful analysis of this finding.

Oncologists frequently cite patient financial considerations as barriers to trial enrollment (28,29). Even among patients able to overcome such barriers, families of children with cancer experience additional financial burdens during and after completion of therapy (30-32), and long-term impacts on families remain a concern across all age groups (33-35). Added financial burdens of travel to and from a CAR-T therapy center coupled with loss of income can exacerbate this economic hardship. It is plausible that oncologists, envisioning the additional acute and chronic toll these stressors impose on families of low SES, may, with good intention, consider such socioeconomic factors when considering referral for CAR-T therapy. Though families may consider goals of care when evaluating clinical options, however, socioeconomics should be considered by the family and care team together after referral so that families can make informed decisions. Even if well intentioned, oncologists making referral decisions based on socioeconomic factors can result in inequities. This study identifies some (albeit subtle) implicit bias in patient scenarios, which is particularly concerning because this has not often been found in other similar studies (36), though we caution against overinterpretation of this finding, given that it was not seen across other comparisons in our hypothetical scenarios. Perhaps more importantly, many respondents indicated a more explicit bias, identifying socioeconomic factors as significantly influential in the referral process. There are few supportable reasons for clinicians to base referral decisions on socioeconomic factors. Although this study alone cannot evaluate the role of physician bias in referral, these findings are concerning and warrant further research to identify whether discriminatory referral practices indeed are taking place.

Although this study did not directly aim to find solutions to these challenges, some findings may be instructive. Establishing formal partnerships with CAR-T therapy centers, for example, may help streamline the referral process. The decision to recommend CAR-T therapy for a patient with relapsed-recurrent B-ALL is variable across pediatric oncology centers, and an established relationship with a CAR-T therapy center can allow for a trusted second opinion for challenging clinical cases. In our study, oncologists at smaller centers were less likely to express familiarity with CAR-T therapy. Many pediatric CAR-T products are offered in clinical trials, but lack of awareness regarding available trials, lack of knowledge of trial details, excessive paperwork, and limited time are known physician barriers to referral to clinical trials (13,37-40). More structured partnerships with CAR-T therapy centers may address many of these barriers by facilitating treatment discussions for challenging clinical cases, identifying patients who may be eligible for CAR-T therapy, and facilitating care coordination. CAR-T therapy centers may have additional resources (financial and otherwise) allocated for these therapies that can help reduce burdens on referring oncologists. Future efforts to improve referral for cellular therapy should include establishment of formal relationships to streamline the referral process.

Improved communication after CAR-T therapy could further strengthen relationships across centers and improve referral. Pediatric patients are often referred to subspecialty services at tertiary-care centers, with limited communication back when patients return to the care of their primary clinicians. Given that there has been little published guidance to date on monitoring for late effects after CAR-T therapy and even less on experimental clinical trial products, oncologists who resume care of patients after CAR-T therapy may benefit from improved communication and guidance from CAR-T therapy centers.

Our study findings should be interpreted in the context of several limitations. First, this pilot study had a small sample size, precluding further detailed analyses. The qualitative sample size was also small, and though interview responses cannot be generalized beyond the studied cohort, inclusion of these perspectives allowed us to contextualize a subset of participants’ quantitative responses. In addition, our study population included only oncologists who had successfully referred patients for previous CAR-T therapy trials. Thus, we were unable to evaluate the perspectives of oncologists who have not previously referred patients or of those who experienced such significant barriers that they could not successfully refer, a population that may differ from the oncologists queried here. Failing to include these perspectives, however, only makes understanding the barriers experienced by individuals who were able to overcome them that much more important. In addition, oncologist demographics were missing in 27% of survey responses, which may limit interpretation of reported findings; however, in sensitivity analysis, there were no statistically significant differences in other survey responses between oncologists who provided demographic data and oncologists with missing demographics. Finally, social acceptability bias may have affected survey responses, though this would have led to an underestimation of the true effect of socioeconomic factors (eg, toward the null).

Despite these limitations, our study provides a valuable early step in understanding referral practices of oncologists for pediatric CAR-T therapy. Recent work has highlighted that unlike standard upfront chemotherapy, outcomes are similar regardless of race, ethnicity, or SES among pediatric patients who receive CAR-T therapy (8,9). Therefore, inclusion of diverse patient populations and improving access to CAR-T therapy are paramount to reducing disparities; optimizing referral by oncologists—including minimization of biases in the referral process—is a key step in achieving this goal. Future efforts to improve access should consider structured partnerships with referring sites to improve access for patients.

## Supplementary Material

pkae063_Supplementary_Data

## Data Availability

The data that support the findings of this study are available from the corresponding author upon reasonable request.
