# Supplementary material for: Perspectives of pediatric oncologists on referral for CAR-T therapy: a mixed methods pilot study
Source: JNCI Cancer Spectr. 2024 Jul 30;8(4):pkae063. doi: 10.1093/jncics/pkae063 (PMC11340642; doi:10.1093/jncics/pkae063)
Supplement: pkae063_Supplementary_Data [file pkae063_supplementary_data.pdf]

Referring Patients for CAR-T Cell Therapy

We appreciate your work and collaboration in pediatric and young adult patients with relapsed/refractory pre-B ALL. This survey is being sent to providers who have referred to our institutions for a CAR-T clinical trial. We are attempting to determine the process of referral and what barriers may be encountered at the provider or patient/caregiver level which may impact the referral process so that we can work on addressing gaps with future CAR-T trials. The survey should take 10-15 minutes to complete. We will present you with 4 scenarios and ask 18 additional questions. The survey is anonymous. There is an opportunity to participate in a follow up interview, though your contact information will not be connected to your survey responses. If there are questions about the survey, please email Adam Lamble at adam.lamble@seattlechildrens.org.

Please complete the survey below.

Thank you!

Clinical Scenario #1:
You are the primary oncologist for a 12-year-old White patient with CD19+ ALL who has relapsed after bone marrow transplant. The patient has tolerated treatment well to date without any current organ dysfunction. The family has expressed a desire to pursue cancer-directed treatment with curative intent, and you are trying to determine the next treatment approach including:
1) referral for CAR-T (Kymriah or clinical trial, potentially followed by bone marrow transplant)
2) reinducing with chemotherapy and/or blinatumomab and second transplant once in remission

Both parents have completed college. They are very familiar with CAR-T therapy.

Based on the information provided:

How medically favorable of a candidate is this patient for CAR-T therapy?

- ◯ Very favorable
- ◯ Somewhat favorable
- ◯ Neither favorable nor unfavorable
- ◯ Somewhat unfavorable
- ◯ Very unfavorable

How likely would you be to refer this patient for CAR-T therapy?

- ◯ Very likely
- ◯ Somewhat likely
- ◯ Neither likely nor unlikely
- ◯ Somewhat unlikely
- ◯ Very unlikely

How likely do you think it is that this patient and family will choose to proceed with CAR-T therapy?

- ◯ Very likely
- ◯ Somewhat likely
- ◯ Neither likely nor unlikely
- ◯ Somewhat unlikely
- ◯ Very unlikely

Cancer Center Experience:
The next 7 questions review your center's experience with CAR-T cell therapies.

What is the number of pediatric oncologists in your group/at your center?
- ◯ Less than 5
- ◯ 5-10
- ◯ Greater than 10

What is the approximate number of new pediatric oncology diagnoses per year at your center?
- ◯ Less than 25
- ◯ 25-50
- ◯ 51-100
- ◯ Greater than 100

How familiar are you with CAR-T cell therapies?
- ◯ Very familiar
- ◯ Somewhat familiar
- ◯ Not familiar

How comfortable are you with providing information regarding CAR-T therapy to your patients and families (i.e., therapeutic options, process for enrollment/administration, potential side effects)?
- ◯ Very comfortable
- ◯ Somewhat comfortable
- ◯ Not very comfortable

In the past 12 months, please estimate how many patients at your center were potentially eligible for a CAR-T product and/or CAR-T clinical trial:
- ◯ 0-1
- ◯ 2-3
- ◯ Greater than 3

In the past 12 months, please estimate what percentage of the potentially eligible patients were referred for a CAR-T product and/or CAR-T clinical trial:
- ◯ 0%
- ◯ 1% to 50%
- ◯ 51% to 99%
- ◯ 100%

Patients or their families may decide against a CAR-T therapy. In the past 12 months, please estimate how many patients/families declined referral for a CAR-T product and/or CAR-T clinical trial:
- ◯ 0-1
- ◯ 2-3
- ◯ Greater than 3

Clinical Scenario #2:
You are the primary oncologist for a 12-year-old Black patient with CD19+ ALL who has relapsed after bone marrow transplant. The patient has tolerated treatment well to date without any current organ dysfunction. The family has expressed a desire to pursue cancer-directed treatment with curative intent, and you are trying to determine the next treatment approach including:
1) referral for CAR-T (Kymriah or clinical trial, potentially followed by bone marrow transplant)
2) reinducing with chemotherapy and/or blinatumomab and second transplant once in remission

Both parents have completed college. They are very familiar with CAR-T therapy.

Based on the information provided:

How medically favorable of a candidate is this patient for CAR-T therapy?
- ◯ Very favorable
- ◯ Somewhat favorable
- ◯ Neither favorable nor unfavorable
- ◯ Somewhat unfavorable
- ◯ Very unfavorable

How likely would you be to refer this patient for CAR-T therapy?
- ◯ Very likely
- ◯ Somewhat likely
- ◯ Neither likely nor unlikely
- ◯ Somewhat unlikely
- ◯ Very unlikely

How likely do you think it is that this patient and family will choose to proceed with CAR-T therapy?

◯ Very likely
◯ Somewhat likely
◯ Neither likely nor unlikely
◯ Somewhat unlikely
◯ Very unlikely

CAR-T Referral:
The next 2 questions aim to understand your CAR-T referral process.

Assuming a patient is clinically eligible for CAR-T therapy, how influential are the following factors in your choice to refer for CAR-T therapy?

	Not influential	Slightly influential	Moderately influential	Significantly influential
Patient/caregiver goals of care	◯	◯	◯	◯
Patient/caregiver interest in CAR-T therapy	◯	◯	◯	◯
Patient/caregiver understanding of CAR-T therapy	◯	◯	◯	◯
Patient/caregiver hesitancy regarding clinical trials	◯	◯	◯	◯
Compliance/adherence concerns	◯	◯	◯	◯
Patient/caregiver resources (i.e., housing, transport, ability to pay bills, etc.)	◯	◯	◯	◯
Family social support	◯	◯	◯	◯
Insurance status	◯	◯	◯	◯
Patient race/ethnicity	◯	◯	◯	◯
Patient/caregiver language and/or cultural barriers	◯	◯	◯	◯
Patient/caregiver education level	◯	◯	◯	◯
Referring institutional support	◯	◯	◯	◯
Support from enrolling site	◯	◯	◯	◯
Other (please explain)	◯	◯	◯	◯

Other: please describe ______________________________

Assuming a patient is clinically eligible for CAR-T therapy, which of the following factors most influences your choice of whether to refer for CAR-T therapy?

- ◯ Patient/caregiver goals of care
- ◯ Patient/caregiver interest in CAR-T therapy
- ◯ Patient/caregiver understanding of CAR-T therapy
- ◯ Patient/caregiver hesitancy regarding clinical trials
- ◯ Compliance/adherence concerns
- ◯ Patient/caregiver resources (i.e., housing, transport, ability to pay bills, etc.)
- ◯ Family social support
- ◯ Insurance status
- ◯ Patient race/ethnicity
- ◯ Patient/caregiver language and/or cultural barriers
- ◯ Patient/caregiver education level
- ◯ Referring institutional support
- ◯ Support from enrolling site
- ◯ Other (please explain)

Other: please describe ______________________________

Clinical Scenario #3:
You are the primary oncologist for a 12-year-old Black patient with CD19+ ALL who has relapsed after bone marrow transplant. The patient has tolerated treatment well to date without any current organ dysfunction. The family has expressed a desire to pursue cancer-directed treatment with curative intent, and you are trying to determine the next treatment approach including:
1) referral for CAR-T (Kymriah or clinical trial, potentially followed by bone marrow transplant)
2) reinducing with chemotherapy and/or blinatumomab and second transplant once in remission

Neither parent completed high school. They are very familiar with CAR-T therapy.

Based on the information provided:

How medically favorable of a candidate is this patient for CAR-T therapy?	◯ Very favorable<br>◯ Somewhat favorable<br>◯ Neither favorable nor unfavorable<br>◯ Somewhat unfavorable<br>◯ Very unfavorable
How likely would you be to refer this patient for CAR-T therapy?	◯ Very likely<br>◯ Somewhat likely<br>◯ Neither likely nor unlikely<br>◯ Somewhat unlikely<br>◯ Very unlikely
How likely do you think it is that this patient and family will choose to proceed with CAR-T therapy?	◯ Very likely<br>◯ Somewhat likely<br>◯ Neither likely nor unlikely<br>◯ Somewhat unlikely<br>◯ Very unlikely

Potential Barriers:
The next 4 questions review potential barriers for patients/families in accessing CAR-T therapy.

Among patients you have referred for CAR-T therapy (via commercially available product or clinical trials) who have encountered barriers to accessing CAR-T, how large do you perceive each of these barriers to be for patients/families?

	Not a barrier at all	A small barrier	A moderate barrier	A large barrier
Patient clinical factors	◯	◯	◯	◯
Patient/caregiver goals of care	◯	◯	◯	◯
Patient/caregiver interest in CAR-T therapy	◯	◯	◯	◯
Patient/caregiver understanding of CAR-T therapy	◯	◯	◯	◯
Patient/caregiver hesitancy regarding clinical trials	◯	◯	◯	◯
Compliance/adherence concerns	◯	◯	◯	◯
Patient/caregiver resources (i.e., housing, transport, ability to pay bills, etc.)	◯	◯	◯	◯
Family social support	◯	◯	◯	◯
Insurance status	◯	◯	◯	◯
Patient race/ethnicity	◯	◯	◯	◯
Patient/caregiver language and/or cultural barriers	◯	◯	◯	◯
Patient/caregiver education level	◯	◯	◯	◯
Referring institutional support	◯	◯	◯	◯
Support from enrolling site	◯	◯	◯	◯
Other (please explain)	◯	◯	◯	◯

Other: please describe ______________________________

Among patients you have referred for CAR-T therapy (via commercially available product or clinical trials) who have encountered barriers to accessing CAR-T, how common do you perceive each of these barriers to be for patients/families?

	Not common at all	A little common	Somewhat common	Very common
Patient clinical factors	◯	◯	◯	◯
Patient/caregiver goals of care	◯	◯	◯	◯
Patient/caregiver interest in CAR-T therapy	◯	◯	◯	◯
Patient/caregiver understanding of CAR-T therapy	◯	◯	◯	◯
Patient/caregiver hesitancy regarding clinical trials	◯	◯	◯	◯
Compliance/adherence concerns	◯	◯	◯	◯
Patient/caregiver resources (i.e., housing, transport, ability to pay bills, etc.)	◯	◯	◯	◯
Family social support	◯	◯	◯	◯
Insurance status	◯	◯	◯	◯
Patient race/ethnicity	◯	◯	◯	◯
Patient/caregiver language and/or cultural barriers	◯	◯	◯	◯
Patient/caregiver education level	◯	◯	◯	◯
Referring institutional support	◯	◯	◯	◯
Support from enrolling site	◯	◯	◯	◯
Other (please explain)	◯	◯	◯	◯

Other: please describe

____

Sometimes the healthcare team can address barriers to accessing CAR-T trials or products. In your experience, what are methods utilized by the healthcare team to overcome such barriers?

____

In your experience, what are methods used by families to overcome such barriers to accessing CAR-T trials or products?

____

Clinical Scenario #4:
You are the primary oncologist for a 12-year-old White patient with CD19+ ALL who has relapsed after bone marrow transplant. The patient has tolerated treatment well to date without any current organ dysfunction. The family has expressed a desire to pursue cancer-directed treatment with curative intent, and you are trying to determine the next treatment approach including:
1) referral for CAR-T (Kymriah or clinical trial, potentially followed by bone marrow transplant)
2) reinducing with chemotherapy and/or blinatumomab and second transplant once in remission

Neither parent completed high school. They are very familiar with CAR-T therapy.

Based on the information provided:

How medically favorable of a candidate is this patient for CAR-T therapy?	◯ Very favorable<br>◯ Somewhat favorable<br>◯ Neither favorable nor unfavorable<br>◯ Somewhat unfavorable<br>◯ Very unfavorable
How likely would you be to refer this patient for CAR-T therapy?	◯ Very likely<br>◯ Somewhat likely<br>◯ Neither likely nor unlikely<br>◯ Somewhat unlikely<br>◯ Very unlikely
How likely do you think it is that this patient and family will choose to proceed with CAR-T therapy?	◯ Very likely<br>◯ Somewhat likely<br>◯ Neither likely nor unlikely<br>◯ Somewhat unlikely<br>◯ Very unlikely

The final 5 questions are clinician demographics.	
What is your age?	◯ Less than 40 years<br>◯ 40-60 years<br>◯ Greater than 60 years
What is your gender?	◯ Male<br>◯ Female<br>◯ Transgender male<br>◯ Transgender female<br>◯ Gender variant/non-conforming<br>◯ Other<br>◯ Prefer not to answer
What is your race? Please check all that apply:	☐ White<br>☐ Black or African American<br>☐ Native American<br>☐ Asian or Asian American<br>☐ Native Hawaiian or Pacific Islander<br>☐ Other<br>☐ Prefer not to answer
What is your ethnicity?	◯ Hispanic<br>◯ Non-Hispanic<br>◯ Prefer not to answer
How many years have you been in practice since completion of pediatric hematology/oncology fellowship training?	◯ Less than 5 years<br>◯ 5-10 years<br>◯ 11-20 years<br>◯ Greater than 20 years

Referring Provider Interview Guide

Hello, this is *[NAME]* from Seattle Children's. We spoke before about participating in an interview, is now still a good time for you?

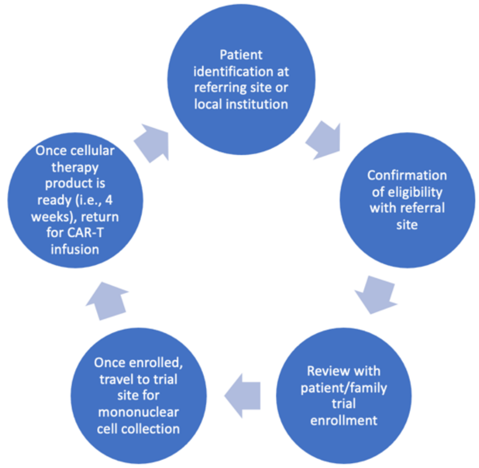

Thanks again for taking the time to talk with me. Today, I'd like to talk with you about referring a patient and family for CAR-T cell therapy, both clinical trials as well as Kymriah, the FDA approved Novartis product. Specifically, I am interested in learning about factors you consider in referring a patient for a CAR-T product as well as the process to do so. We are trying to better understand where there may be gaps in this process which impede referrals and what are barriers for patients and families that we can hope to better address.

Does this all sound okay to you?

Before we begin, I'd like to review a few important points:

- The purpose of this project is to understand your perspective and experience. Please share exactly what you think and don't worry about what I think.

You are welcome to skip any question or to request that we finish the interview early for whatever reason.

[Permission to record]

Can you confirm for the recording that we have your permission to record?

Do you have any questions for me before we get started?

1. As a starting point, please tell me your role within hem/onc/BMT and in your department/division as well as a little more about your hospital setting.

Referral decision making:

First, we are trying to understand what information regarding CAR-T theargy is important and available for referring providers and their patients. Consider a scenario in which CAR-T would be an option for your patient:

2. Tell us about your decision making process and discussion with the family when CAR-T is an option.

- Do you typically discuss other therapeutic options?
- If so, what other options do you discuss?
- What would make you discuss other therapeutic options?
- What helps you and a family decide between these options?

3. As a referring provider for CAR-T, where do you obtain most of your information regarding CAR-T therapies?
4. In our survey responses, it appears there are gaps in how comfortable some referring providers are about CAR-T—do you have any suggestions about how to improve education about CAR-T therapies (including what clinical trials are available?)

Second, we are trying to understand patient/family-level factors which may be impacting the referral process:

5. In our survey responses, many patients/families that were eligible for CAR-T were not referred. We understand many factors go into the referral process for CAR-T. Has this been your experience?
- Why do you think some patients may not have been referred?
- Or were they referred for a different treatment/trial, and if so why?
6. In many of the survey responses we received, patient/family understanding of and interest in CAR-T therapies was moderately or significantly influential in the decision to refer a patient. What has your experience been?
7. How do a patient and family's access to certain resources impact the likelihood that you would recommend CAR-T?

Barriers:

Third, we are trying to understand and address barriers for families in understanding and accessing CAR-T therapies

8. What are some of the barriers patients and families experience when trying to access CAR-T therapy?
- Primary language
- Economic resources (housing, travel)
- Social support (other children, ageing parents, single parent)
- Insurance status
9. In your experience, what has helped families overcome these barriers?
10. Are there resources that you think are vital for the referring institution (i.e., your institution) to provide if you are referring a patient out?
11. What about the enrolling site?
12. Can you tell me about a situation where lack of resources inhibited a family from accessing CAR-T therapy?
13. Would the availability of a local therapy overcome these barriers?
14. Can you think of any reasons why a patient/family may decline CAR-T?

15. Is there anything else you would like to share about your experience?

That was my last question. Do you have anything you'd like to add, or any questions for me?

Thank you again for taking the time to talk with me. We appreciate your willingness to share your experience and your thoughts.
